# Impact of cell cycle on repair of ruptured nuclear envelope and sensitivity to nuclear envelope stress in glioblastoma

**DOI:** 10.1038/s41420-023-01534-7

**Published:** 2023-07-08

**Authors:** Yasunao Kamikawa, Zuqian Wu, Nayuta Nakazawa, Taichi Ito, Atsushi Saito, Kazunori Imaizumi

**Affiliations:** Department of Biochemistry, Institute of Biomedical & Health Sciences, Hiroshima University, 1-2-3 Kasumi, Minami-ku, Hiroshima, 734-8553 Japan

**Keywords:** Cell biology, Molecular biology

## Abstract

The nuclear envelope (NE) is often challenged by various stresses (known as “NE stress”), leading to its dysfunction. Accumulating evidence has proven the pathological relevance of NE stress in numerous diseases ranging from cancer to neurodegenerative diseases. Although several proteins involved in the reassembly of the NE after mitosis have been identified as the NE repair factors, the regulatory mechanisms modulating the efficiency of NE repair remain unclear. Here, we showed that response to NE stress varied among different types of cancer cell lines. U251MG derived from glioblastoma exhibited severe nuclear deformation and massive DNA damage at the deformed nuclear region upon mechanical NE stress. In contrast, another cell line derived from glioblastoma, U87MG, only presented mild nuclear deformation without DNA damage. Time-lapse imaging demonstrated that repairing of ruptured NE often failed in U251MG, but not in U87MG. These differences were unlikely to have been due to weakened NE in U251MG because the expression levels of lamin A/C, determinants of the physical property of the NE, were comparable and loss of compartmentalization across the NE was observed just after laser ablation of the NE in both cell lines. U251MG proliferated more rapidly than U87MG concomitant with reduced expression of p21, a major inhibitor of cyclin-dependent kinases, suggesting a correlation between NE stress response and cell cycle progression. Indeed, visualization of cell cycle stages using fluorescent ubiquitination-based cell cycle indicator reporters revealed greater resistance of U251MG to NE stress at G_1_ phase than at S and G_2_ phases. Furthermore, attenuation of cell cycle progression by inducing p21 in U251MG counteracted the nuclear deformation and DNA damage upon NE stress. These findings imply that dysregulation of cell cycle progression in cancer cells causes loss of the NE integrity and its consequences such as DNA damage and cell death upon mechanical NE stress.

## Introduction

The nuclear envelope (NE) encloses genomic DNA to protect it from a variety of insults and plays pivotal roles in regulating genomic function [1, 2]. Previous studies demonstrated that the NE is damaged upon various types of stresses, leading to its dysfunction [3–7]. Such stresses are referred to as “NE stress” [8–11]. Mechanical NE stress frequently results in the rupture of the NE [3, 4]. Cell migration through confined environments is one of the major causes of NE rupture [3, 4]. This can include both physiological and pathological events such as the radial migration of neural progenitors during brain development and the invasion of tumor cells into surrounding tissues, respectively [3, 4, 12]. Accumulating evidence has revealed that NE rupture is involved in a broad spectrum of diseases ranging from cancer to neurodegenerative diseases. Regarding the pathology of cancer, NE rupture has been proposed to trigger genome instability and increased cellular invasion through DNA damage [13, 14]. Mechanistically, studies have suggested that the mislocalization of cytoplasmic DNase and/or nuclear DNA repair factors is responsible for such DNA damage [14, 15].

Time-lapse imaging has revealed that most NE rupture is repaired within an hour, indicating the presence of machinery to carry out such repair [3, 4, 16–18]. Indeed, several factors involved in repairing ruptured NE have been identified, including a small DNA binding protein, Barrier-to-autointegration factor (BAF), and its binding partners, Lap2-emerin-MAN1 (LEM) domain proteins, which integrate into the inner nuclear membrane [16, 17]. It has been proposed that LEM domain proteins further recruit endosomal sorting complex required for transport III (ESCRT III), which mediates the resealing of ruptured NE [3, 4, 16, 19].

Despite the crucial roles of these factors in repairing ruptured NE, dysregulation of their activities potentially disrupts the integrity of the NE. In vitro functional analysis has demonstrated that hyperactivation of ESCRT III results in the loss of integrity of the NE [20]. In addition, the aberrant nuclear accumulation of VPS4, which is the ATPase for ESCRT III and involved in ESCRT III-dependent membrane remodeling, has been observed in the neurons in both familiar and sporadic amyotrophic lateral sclerosis patients [21]. These findings suggest that the response to NE stress needs to be fine-tuned in a cell-type-specific manner, although the underlying mechanisms have yet to be described. In higher eukaryotes, the NE is disassembled and reassembled before and after mitosis, respectively [22]. During these processes, the factors involved in NE repair drastically changes their localization and play pivotal roles in remodeling of the NE structure. As in NE repair, the sequential recruitment of BAF, LEM domain proteins, and ESCRT III is indispensable for the reassembly of the NE [22, 23]. In contrast, prior to the disassembly of the NE, the loss of interaction between BAF and DNA leads to the dispersal of the LEM domain proteins to the endoplasmic reticulum and further contributes to the removal of membranes from chromosomes [22]. The spatiotemporal distributions of BAF, LEM domain proteins, and ESCRT III are determined by their phosphorylation status, which is mainly mediated by the cell-cycle-dependent activation of various protein kinases, including cyclin-dependent kinase 1 (CDK1) and vaccinia-related kinase 1 [22, 24, 25]. Together with the mechanistic similarity between repair and reassembly of the NE, these findings imply that NE repair is also regulated in a manner dependent on cell cycle progression.

In the present study, we investigated the cellular response to mechanical NE stress and found that the NE stress response varies among different types of cancer cell lines. Our data further demonstrated that cell cycle progression correlated with the sensitivity to NE stress due to compromised NE repair at the specific stages of the cell cycle in some glioblastoma cell lines. These findings shed light on the unexpected complexity of cellular response to mechanical NE stress and its underlying mechanism.

## Results

### Cell-type-specific response to mechanical NE stress

**Fig. 1:**
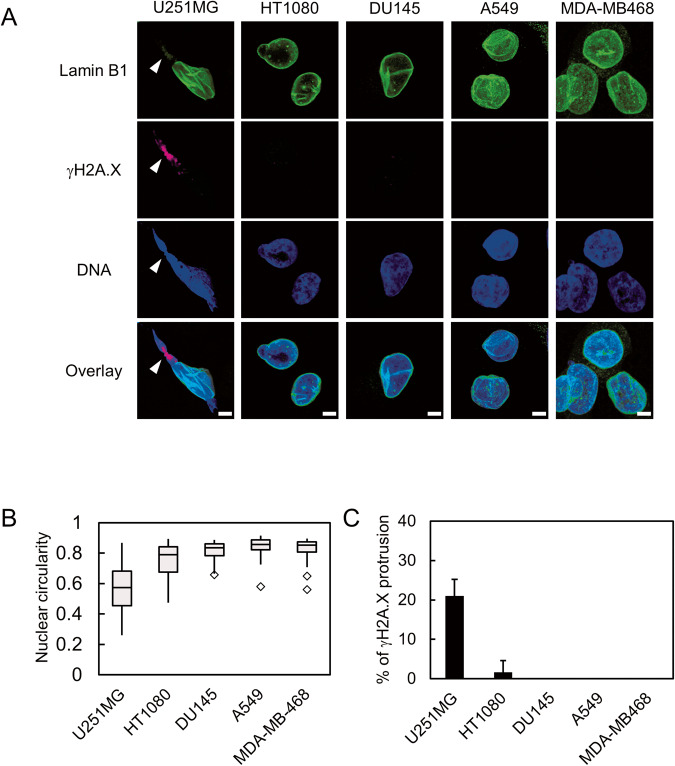
Response to mechanical NE stress varies among cancer cell lines. **A** Immunofluorescence staining analysis of lamin B1 (green) and γH2A.X (magenta) of U251MG, HT1080, DU145, A549, and MDA-MB468 cells on the bottom side of a Transwell with 3 μm pore size. Overlay indicates merged images with DNA (blue) staining. Arrowheads represent the nuclear protrusion of U251MG, where lamin B1 is depleted while γH2A.X is accumulated. Scale bars: 5 μm. **B** Quantification of nuclear circularity of the cell lines indicated in (**A**). Box plots represent the medians and interquartile ranges with Tukey-style whiskers. Total cell numbers for each cell line from three independent experiments: *n* = 48, U251MG; *n* = 66, HT1080; *n* = 22, DU145; *n* = 36, A549; and *n* = 37, MDA-MB468. **C** Quantification of the cells with γH2AX accumulation at the nuclear protrusions indicated in (**A**). Bars and error bars represent the mean values and standard deviations from three independent experiments. Total cell numbers for each cell line from three independent experiments: *n* = 47, U251MG; *n* = 66, HT1080; *n* = 22, DU145; *n* = 36, A549; and *n* = 37, MDA-MB468.

To investigate the differential response to mechanical NE stress among cancer cell lines, five cell lines (U251MG, HT1080, DU-145, A549, and MDA-MB468) derived from different types of cancers [glioblastoma (GBM), fibrosarcoma, prostate cancer, non-small cell lung cancer, triple-negative breast cancer] were plated on a Transwell, a porous membrane chamber with a pore size of 3 μm. The nuclei of cells migrating through the Transwell are confined, exposing them to substantial mechanical NE stress [9, 15]. Here, the cells on the bottom side of the Transwell were subjected to immunostaining using antibodies against lamin B1 and γH2A.X, which are markers of the nuclear lamina and DNA double strand breaks, respectively [9, 15]. Previous studies reported that NE stress causes partial disruption of the nuclear lamina and increases DNA damage [9, 26, 27]. We found that some of the U251MG cells exhibited severe nuclear deformation with protrusion(s) extending out of the nucleus (Fig. 1A, arrowheads). Such deformation was hardly observed in other cell lines. Consistent with this, the nuclear circularity was lowest in U251MG among all of the tested cell lines (Fig. 1B). The signal of lamin B1 was partially depleted from the deformed region of the nuclei of U251MG cells, suggesting local disruption of the nuclear lamina (Fig. 1A). Additionally, γH2A.X specifically accumulated in the nuclear protrusions, where signal of lamin B1 is depleted, in approximately 20% of U251MG cells (Fig. 1A and C). Such accumulation of the γH2A.X signal was rarely observed in the nuclei of other types of cells (Fig. 1C). Taken together, these results suggest that U251MG cells are sensitive to mechanical NE stress, leading to DNA damage.

**Fig. 2:**
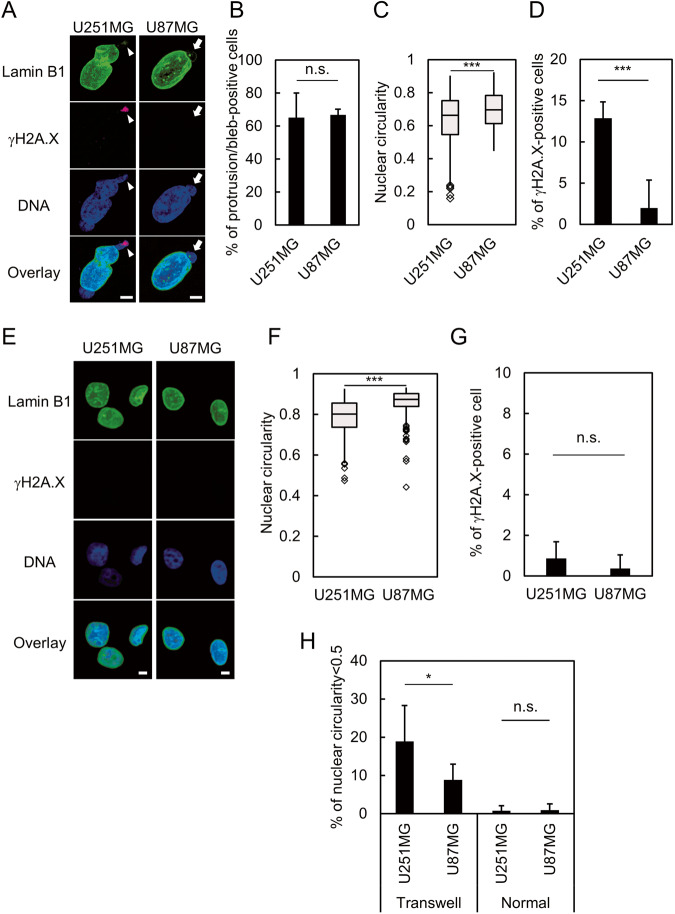
U251MG and U87MG reveal distinct responses to NE stress. **A** Immunofluorescence staining analysis of lamin B1 and γH2A.X of U251MG and U87MG cells on the bottom side of a Transwell. Overlay indicates merged images with DNA (blue) staining. Arrows indicate nuclear bleb. Arrowheads indicate the nuclear protrusion where lamin B1 (green) is depleted while γH2A.X (magenta) is accumulated. Scale bars: 5 μm. **B** Quantification of nuclear protrusion or bleb of U251MG and U87MG cells at the bottom side of the Transwell shown in (**A**). Bars and error bars represent the mean values and standard deviations from three independent experiments. Statistical significance of the difference was determined using Fisher’s exact test. n.s.: *p* > 0.05. Total cell numbers for each cell line from three independent experiments: *n* = 100, U251MG; and *n* = 136, U87MG. **C** Quantification of nuclear circularity of U251MG and U87MG cells at the bottom side of the Transwell shown in (**A**). Box plots represent the medians and interquartile ranges with Tukey-style whiskers from three independent experiments. Statistical significance of the difference was determined using Brunner–Munzel test. ***p < 0.005. Total cell numbers for each cell line from three independent experiments: *n* = 210, U251MG; and *n* = 136, U87MG. **D** Quantification of the cells with γH2A.X accumulation at the nuclear protrusion or bleb in U251MG and U87MG cells at the bottom side of the Transwell shown in (**A**). Bars and error bars represent the mean values and standard deviations from three independent experiments. Statistical significance of the difference was determined using Fisher’s exact test. ***p < 0.005. Total cell numbers for each cell line from three independent experiments: *n* = 218, U251MG; and *n* = 136, U87MG. **E** Immunofluorescence staining analysis of lamin B1 (green) and γH2A.X (magenta) of U251MG and U87MG cells without NE stress. Overlay indicates merged images with DNA (blue) staining. Scale bars: 5 μm. **F** Quantification of nuclear circularity of U251MG and U87MG cells without NE stress indicated in (**E**). Box plots represent the medians and interquartile ranges with Tukey-style whiskers from three independent experiments. Statistical significance of the difference was determined using Brunner–Munzel test. ***p < 0.005. Total cell numbers for each cell line from three independent experiments: *n* = 258, U251MG; and *n* = 179, U87MG. **G** Quantification of the cells with γH2A.X accumulation at the nuclear protrusion in U251MG and U87MG cells without NE stress indicated in (**E**). Bars and error bars represent the mean values and standard deviations from three independent experiments. Statistical significance of the difference was determined using Fisher’s exact test. n.s.: *p* > 0.05. Total cell numbers for each cell line from three independent experiments: *n* = 285, U251MG; and *n* = 195, U87MG. **H** Quantification of severely deformed nuclei (circularity < 0.5) in U251MG and U87MG cells with (Transwell) or without (Normal) NE stress indicated in (**A**) and (**E**). Bars and error bars represent the mean values and standard deviations from three independent experiments. Statistical significance of the difference was determined using Fisher’s exact test. *p < 0.05. n.s.: *p* > 0.05. Total cell numbers for each cell line and condition from three independent experiments: *n* = 210, U251MG Transwell; *n* = 136, U87MG Transwell; *n* = 258, U251MG normal; and *n* = 179, U87MG normal.

It has been proposed that nuclear deformability correlates with the ability of cells to migrate through confined environments [28]. Given that GBM is one of the most invasive cancer types, we asked whether sensitivity to mechanical NE stress is shared among GBM. To test this, we compared U251MG with another GBM-derived cell line, U87MG, using a Transwell. In U87MG, migration through the Transwell resulted in the formation of nuclear blebs in 65% of cells, which are well-known abnormal nuclear structures caused by disorganization of the nuclear lamina (Fig. 2A, B) [26, 27]. Indeed, these nuclear blebs were devoid of lamin B1 signal (Fig. 2A, arrows). However, the nuclear circularity of U87MG was significantly higher than that of U251MG (Fig. 2C). Consistently, the proportions of the cells with severely deformed nuclei (circularity<0.5) was significantly higher in U251MG cells (Fig. 2H). Moreover, the strong signal of γH2A.X at the deformed nuclear region was only observed in U251MG cells (Fig. 2A, arrowheads and Fig. 2D), but not in U87MG (Fig. 2A, arrows and Fig. 2D), suggesting that sensitivity to NE stress is a unique feature of U251MG rather than being common to GBM. Under normal condition, such severely deformed nuclei and the prominent signal of γH2A.X were hardly observed in both U251MG and U87MG cell lines (Fig. 2E–H). Subsequently, we performed a comparison between U251MG and U87MG to identify the mechanisms underlying their differential sensitivity to mechanical NE stress.

**Fig. 3:**
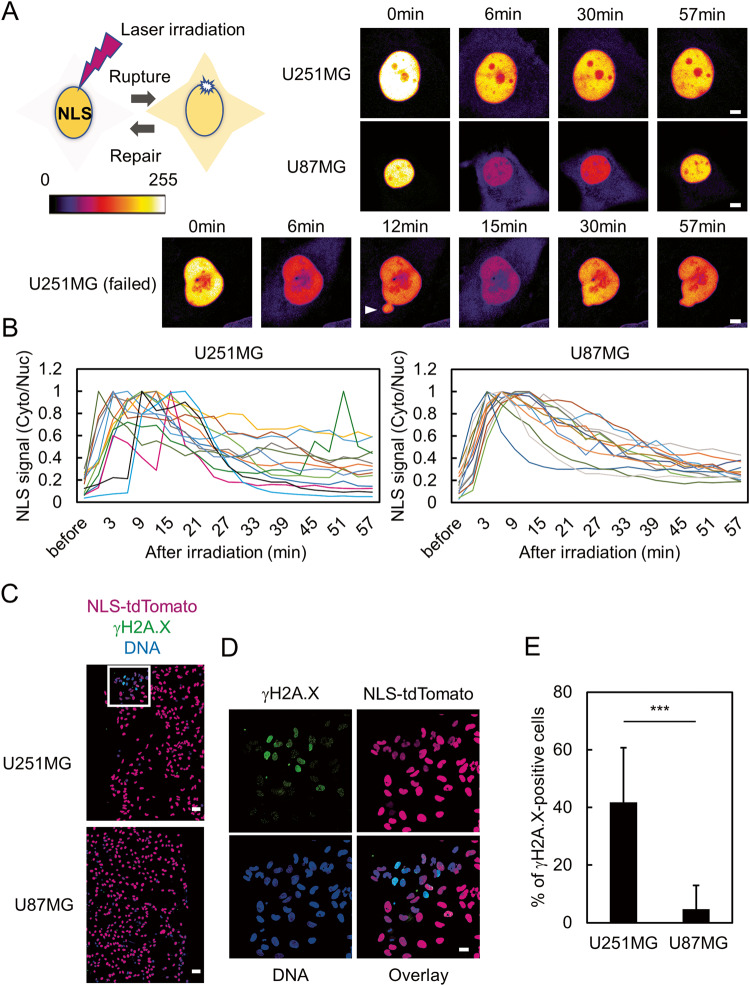
Differential dynamics of repairing ruptured NE between U251MG and U87MG cells. **A** Dynamics of repair of NE rupture induced by laser irradiation in U251MG and U87MG. Left: Schematic presentation of laser ablation to induce NE rupture and monitor NE repair. NLS: nuclear localization signal. Right: Snapshot images of time-lapse imaging of NLS-tdTomato in U251MG and U87MG. Interval and duration were 3 and 57 min, respectively. U251MG (failed) represents a typical cell that formed a nuclear bleb at the site of laser ablation (arrowhead) and experienced additional rupture of the NE. Scale bars: 5 μm. **B** Quantification of the ratio of NLS-tdTomato signal intensity of the cytoplasm to that of the nucleus shown in (**A**). The raw values at the highest time point were set at 1.0. Total cell numbers for each cell line from three independent experiments: *n* = 13, U251MG; and *n* = 13, U87MG. **C** Immunofluorescence staining analysis of γH2A.X (green) of U251MG and U87MG cells after laser irradiation together with NLS-tdTomato (magenta) and DNA (blue). Scale bars: 50 μm. **D** Enlarged images of the region indicated as a square in (**C**). Scale bar: 20 μm. **E** Quantification of the cells positive for prominent γH2A.X signal shown in (**C**). Bars represent the mean values from three independent experiments. Statistical significance of the difference was determined using Fisher’s exact test. ***p < 0.005. Total cell numbers for each cell line from three independent experiments: *n* = 73, U251MG; and *n* = 71, U87MG.

To test whether sensitivity to NE stress is correlated with the efficiency of repairing ruptured NE, we evaluated the dynamics of NE repair in U251MG and U87MG. For this purpose, the fluorescent protein tdTomato fused with a nuclear localization signal (NLS) was stably expressed in U251MG as well as U87MG and was used as a reporter of NE integrity (Fig. 3A). The expression level of NLS-tdTomato was comparable between these cell lines (Supplementary Fig. 1A, B). A small region of the nuclear periphery was irradiated with a 405 nm laser to induce local rupture of the NE (Fig. 3A) [16–18]. We also confirmed the accumulation of mScarlet tagged BAF, which is a factor that accumulates at the site of NE rupture, just after laser irradiation (Supplementary Fig. 2C) [16, 17]. Time-lapse imaging of NLS-tdTomato in U251MG and U87MG was performed after laser ablation and the ratio of cytoplasmic NLS-tdTomato signal to that of the nucleus was determined. Cytoplasmic leakage of NLS-tdTomato was observed in both U251MG and U87MG just after laser irradiation, indicating that their NE was ruptured (Fig. 3A, B). In U87MG, almost all of the tested cells revealed gradual re-accumulation of NLS-tdTomato, representing the repair of ruptured NE in these cells (Fig. 3A, B). In contrast, some of the U251MG cells formed a nuclear bleb at the site of laser irradiation (Fig. 3A, arrowhead). In addition, cytoplasmic NLS-tdTomato signal rapidly increased after the formation of bleb, suggesting additional NE rupture at the nuclear bleb (Fig. 3A, B). These results suggest that the repair of ruptured NE is occasionally failed in U251MG, but not in U87MG. We further tested whether U251MG cells are more sensitive to the NE stress induced by laser irradiation than U87MG cells. U251MG and U87MG cells were subjected to laser irradiation of the NE, and cultured for approximately additional 16 h. Then, the levels of DNA damage was evaluated by γH2A.X. In 42% of irradiated U251MG cells, a prominent signal of γH2A.X was revealed, whereas such increased signal was not observed in U87MG cells (Fig. 3C, D). These results suggested that the differential repair dynamics of ruptured NE at least partially correlate with the sensitivity to NE stress.

### Attenuation of cell cycle progression confers resistance to NE stress

**Fig. 4:**
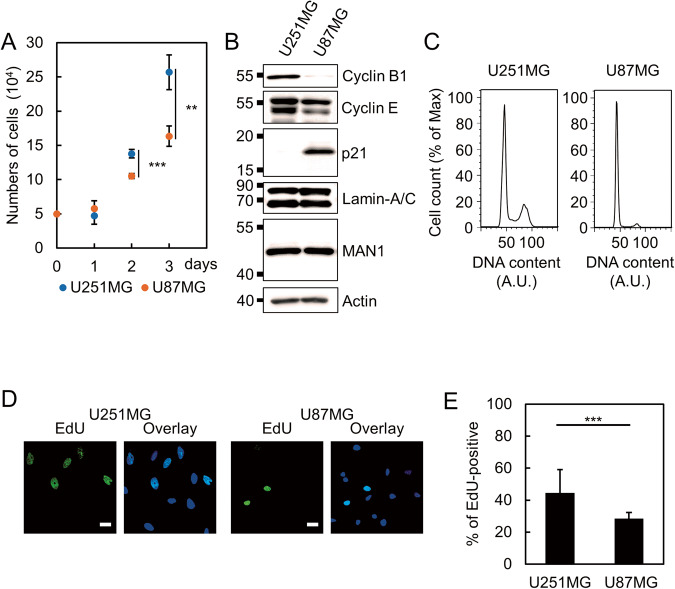
Differential cell cycle progression between U251MG and U87MG cells. **A** The numbers of U251MG and U87MG cells at the indicated time points were represented as median in box plots. Interquartile ranges from three independent experiments were shown as Tukey-style whiskers. Statistical significance of the difference was determined using Student’s *t* test. **p < 0.01. ***p < 0.005. **B** Western blotting analysis of U251MG and U87MG. β-Actin was used as a loading control. **C** DNA content analysis using PI staining followed by flow cytometry. A.U.: arbitrary unit. **D** EdU incorporation assay of U251MG cells and U87MG cells. The cells were incubated with 25 μM EdU for 30 min. S-phase cells were visualized with click reaction. Scale bars: 20 μm. **E** Quantification of EdU-positive cells indicated in (**D**). Bars and error bars represent the mean values and standard deviations from three independent experiments. Statistical significance of the difference was determined using Fisher’s exact test. ***p < 0.005. Total cell numbers for each cell line from three independent experiments: *n* = 268, U251MG; and *n* = 272, U87MG.

Our data suggest that U251MG cells are more sensitive to NE stress than U87MG cells. Thus, we investigated the causes of this difference. We noticed that U251MG had faster cell cycle progression than U87MG (Fig. 4A). Consistently, cyclin E and cyclin B1 were more abundantly expressed in U251MG, whereas the major CDK inhibitor p21 was highly expressed in U87MG (Fig. 4B) [29]. In contrast, the expression levels of lamin A/C and MAN1, which are involved in NE repair, were comparable between U251MG and U87MG (Fig. 4B) [16, 18]. Cytometric analysis of propidium iodide (PI) showed that the proportions of cells in S and G_2_/M phases were higher in U251MG than in U87MG (Fig. 4C). By analyzing the incorporation of ethynyl-deoxyuridine (EdU), we also confirmed that a greater proportion of S-phase cells were in U251MG than in U87MG (Fig. 4D, E).

**Fig. 5:**
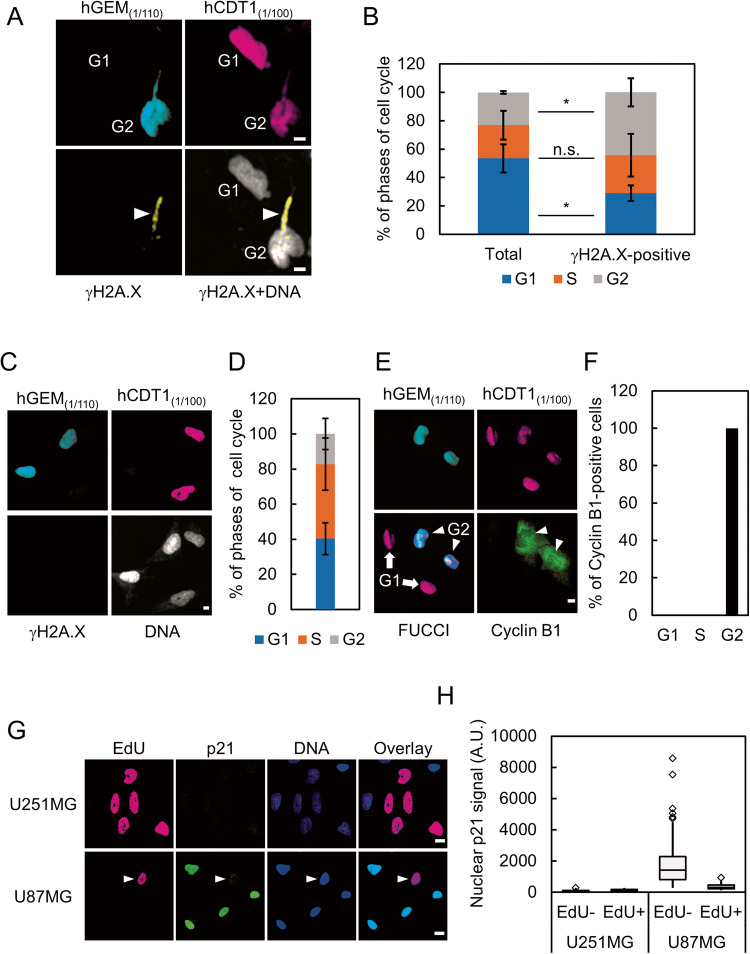
Cell cycle progression correlates with sensitivity to NE stress. **A** Visualization of cell cycle stage using FUCCI reporters, mTurquoise-tagged hGEM_(1/110)_ (cyan) and mCherry-tagged hCdt1_(1/100)_ (magenta). Arrow represents the nuclear protrusion where γH2A.X (yellow) is accumulated. DNA is visualized with DRAQ5 (gray). Scale bars: 5 μm. **B** Quantification of the stages of the cell cycle in U251-FUCCI at the bottom side of the Transwell shown in (**A**). Total: All tested cells at the bottom side of Transwell; γH2AX-positive: The cells with prominent signal of γH2A.X. Statistical significance of the differences was determined using Student’s *t* test from three independent experiments. n.s.: *p* > 0.05 *p < 0.05. Total number for each condition from three independent experiments: *n* = 278, Total; and *n* = 44 γH2A.X-positive. **C** Immunofluorescence staining analysis of γH2A.X (green) in U251-FUCCI cells without NE stress. Scale bar: 5 μm. **D** Quantification of the stages of cell cycle in U251-FUCCI cells without NE stress shown in (**C**). Total number of cells from three independent experiments: *n* = 54. **E** Immunofluorescence staining analysis of cyclin B1 (green) in U251-FUCCI cells. Arrows: G_1_-phase cells. Arrowheads: G_2_-phase cells. Scale bar: 5 μm. **F** Quantification of cyclin B1-positive cells shown in (**E**)*.* Total number of cells from three independent experiments: *n* = 148. **G** Immunofluorescence staining analysis of p21 (green) in U251MG and U87MG cells with visualization of EdU incorporation (magenta). Overlay indicates merged images with DNA (blue) staining. Scale bars: 10 μm. **H** Quantification of relative p21 signal in the nucleus shown in (**G**). Box plots represent the medians and interquartile ranges with Tukey-style whiskers from three independent experiments. Total cell numbers for each cell line: *n* = 83 U251MG EdU-negative; *n* = 64 U251MG EdU-positive; *n* = 74 U87MG EdU-negative, and *n* = 45 U87MG EdU-positive.

Most NE repair factors have been shown to be involved in the reassembly of the NE after mitosis [22, 23]. In this context, the localization of these factors is dynamically regulated in a cell-cycle-dependent manner [22, 24, 25]. Therefore, we tested whether cell cycle progression is also involved in regulating the NE repair pathway and can explain the differential NE stress response between U251MG and U87MG. To achieve this, we stably introduced a fluorescent reporter system for visualizing the stages of the cell cycle, fluorescent ubiquitination-based cell cycle indicator (FUCCI), into U251MG and obtained a cell line designated U251-FUCCI [30]. In this system, the cell cycle-dependent degradation of two protein fragments, hCDT1_(1/100)_ and hGEM_(1/110)_ derived from amino acids 1–100 of hCDT1 and amino acids 1–110 of hGEM, respectively, were used to visualize the stages of the cell cycle. hCDT1 is a key factor for the licensing of DNA replication. To prevent excess DNA replication, its degradation occurs, apart from during G_1_ phase, by the ubiquitin proteasome pathway. hCDT1_(1/100)_ includes the amino acid sequence for the S-phase-specific degradation of hCDT1, enabling cells at G_1_, G_2_, and M phases to be labeled. hGEM is an inhibitor of hCDT1 and is degraded by the proteasome only in G_1_ phase. hGEM_(1/110)_ recapitulates this expression pattern of hGEM, thus enabling the visualization of cells in S, G_2_, and M phases. Therefore, hCDT1_(1/100)_-positive, hGEM_(1/110)_-positive, and hCDT1_(1/100)_-hGEM_(1/110)_-double-positive cells are classified into G_1_, S, and G_2_/M phases, respectively [30]. U251-FUCCI cells were exposed to mechanical NE stress using the Transwell and γH2A.X at the deformed nuclei was evaluated together with the stages of the cell cycle (Fig. 5A). M-phase cells were excluded from the quantification because the NE is disassembled at this stage of the cell cycle. In the entire population of U251-FUCCI cells, G_1_-, S-, and G_2_-phase cells constituted 53%, 23%, and 23% of total cells at the bottom side of the Transwell, respectively (Fig. 5B). The proportion of G_1_-phase cells was significantly decreased to 29% cells with a prominent signal of γH2A.X (Fig. 5B). In contrast, the proportion of G_2_-phase cells among those cells exhibited an approximate doubling (44%) and that of S-phase cells did not differ (Fig. 5B). Under normal conditions, an increased presence of a prominent γH2A.X signal was hardly observed in the G_1_, S, and G_2_-phases of the cell cycle (Fig. 5C, D). To validate the fidelity of FUCCI reporter, we tested the expression of cyclin B1, a marker of G_2_/M-phases, in U251-FUCCI cells without NE stress. Neither hGEM-single-positive nor hCDT1-single-positive cells represented the strong signal of cyclin B1 (Fig. 5E, F). In contrast, hGEM-hCDT1 double-positive cells showed robust signal of cyclin B1 (Fig. 5E, F). Taken together, these results indicate that G_1_-phase cells are more resistant to NE stress than S- and G_2_-phases cells. To test the expression pattern of p21 in U251MG and U87MG, we performed immunostaining of p21 together with the visualization of EdU. As shown in Fig. 5G, H, the signal of nuclear p21 was found to be depleted in S-phase U87MG cells (Fig. 5G, arrowhead and Fig. 5H). In contrast, p21 is greatly reduced in almost all the population of U251MG cells (Fig. 5G, H), suggesting that the depletion of p21 is necessary but not sufficient for the increased sensitivity to mechanical NE stress.

**Fig. 6:**
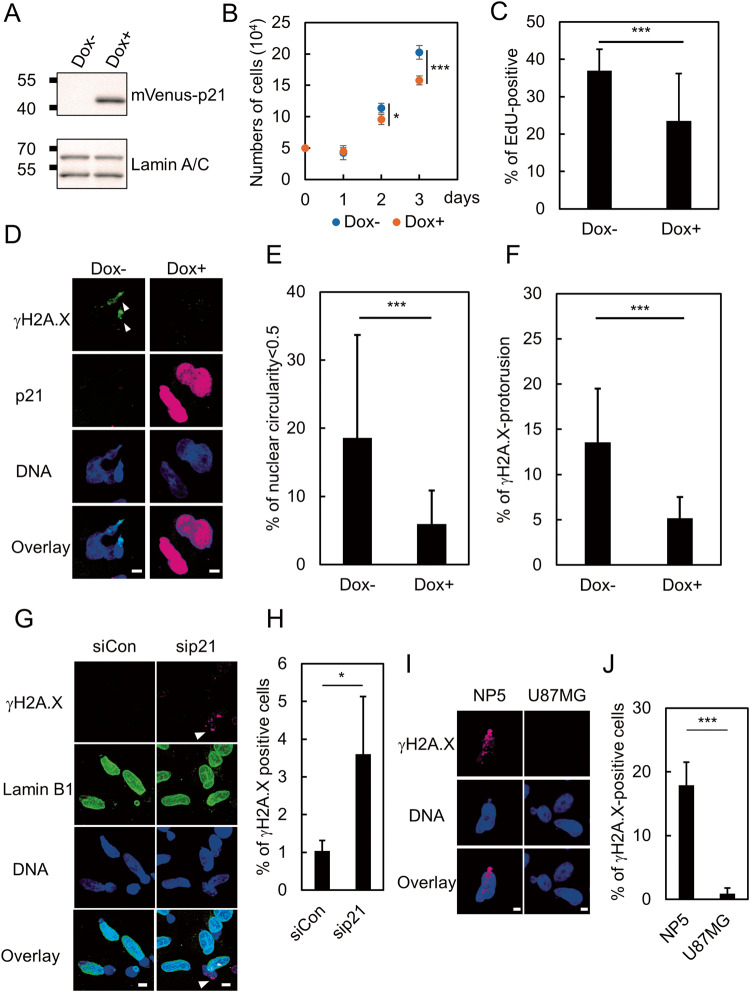
p21 confers resistance to NE stress by restricting cell cycle progression. **A** Western blotting analysis of U251-Tet-p21 in the absence (Dox−) or presence of Dox (Dox+). Lamin A/C were used as loading controls. **B** The numbers of U251-Tet-p21 cells in the absence (Dox−) of or presence of Dox (Dox+). Box plots represent the medians and interquartile ranges with Tukey-style whiskers at the indicated time point from three independent experiments. Statistical significance of the difference was determined using Student’s *t* test. *p < 0.05. ***p < 0.005. **C** Quantification of EdU incorporation of U251-Tet-p21 in the absence (Dox−) or presence of Dox (Dox+). Bars and error bars represent the mean values and standard deviations from three independent experiments. Statistical significance of the difference was determined using Fisher’s exact test. ***p < 0.005. Total cell numbers for each condition from three independent experiments: *n* = 303, Dox−; and *n* = 286, Dox+. **D** Immunofluorescence staining analysis of γH2A.X of U251MG-Tet-p21 in the absence (Dox−) or presence of Dox (Dox+). Arrowheads represent the nuclear protrusion where γH2A.X is accumulated. Scale bars: 5 μm. **E** Quantification of the U251-Tet-p21 cells with severely deformed nucleus (circularity < 0.5). Bars and error bars represent the mean values and standard deviations from three independent experiments. Statistical significance of the difference was determined using Fisher’s exact test. ***p < 0.005. Total cell numbers for each condition from three independent experiments: *n* = 318, Dox−; and *n* = 267, Dox+. **F** Quantification of the cells with γH2A.X accumulation in U251-Tet-p21 cells. Bars and error bars represent the mean values and standard deviations from three independent experiments. Statistical significance of the difference was determined using Fisher’s exact test. ***p < 0.005. Total cell numbers for each condition from three independent experiments: *n* = 354, Dox−; and *n* = 273, Dox+. **G** Immunofluorescence staining analysis of lamin B1 (green) and γH2A.X (magenta) of U87MG cells with (sip21) or without (siCon) RNAi of p21 at the bottom side of a Transwell. Arrowhead indicates the prominent signal of γH2A.X accumulated to the nuclear bleb. Overlay indicates merged images with DNA (blue) staining. Scale bars: 5 μm. **H** Quantification of the cells with γH2AX accumulation at the nuclear bleb shown in (**G**). Bars and error bars represent the mean values and standard deviations from three independent experiments. Statistical significance of the difference was determined using Fisher’s exact test. *p < 0.05. Total cell numbers for each condition from three independent experiments: *n* = 376, siCon; and *n* = 627, sip21. **I** Immunofluorescence staining analysis of γH2A.X and DNA of NP5 and U87MG cells on the bottom side of a Transwell. Scale bars: 5 μm. **J** Quantification of the cells with γH2A.X accumulation at the nuclear protrusion in NP5 and U87MG cells at the bottom side of a Transwell shown in (**I**). Bars and error bars represent the mean values and standard deviations from three independent experiments. Statistical significance of the difference was determined using Fisher’s exact test. ***p < 0.005. Total cell numbers for each cell line from three independent experiments: *n* = 173, NP5; and *n* = 320, U87MG.

To further clarify the relationship between cell cycle progression and NE stress response, we established a cell line that expresses a fusion protein of p21 and mVenus in the presence of doxycycline (Dox) derived from U251MG, designated U251-Tet-p21. We confirmed the induction of mVenus-p21 and the reduction of the total number of cells as well as the proportion of S-phase cells in the absence (Dox−) or presence of Dox (Dox+) (Fig. 6A–C). U251-Tet-p21 cells were cultured in the absence or presence of Dox and then subjected to mechanical NE stress using the Transwell. The sensitivity to NE stress was evaluated by measuring the nuclear circularity as well as the accumulation of γH2A.X in these cells’ nuclear protrusions. As shown in Fig. 6D–F, the proportion of cells with severe nuclear deformation (nuclear circularity < 0.5) and a prominent γH2A.X signal was significantly decreased in the presence of Dox (Fig. 6D–F). In contrast, the depletion of p21 in U87MG by RNAi resulted in a significant increase of γH2A.X positive cells at the bottom side of the Transwell (Fig. 6G, H). Collectively, these results suggest that p21 reinforces the resistance to NE stress by restricting cell cycle progression. To investigate the impact of cell cycle and expression of p21 in the sensitivity to mechanical NE stress, we explored additional GBM cell line in which expression level of p21 and proportion of G_1_-phase cells are relatively lower than in U87MG. We chose NP5 based on the expression level of p21 in public available database, DepMap [31]. Cytometric analysis of PI revealed that the proportions of cells in S and G_2_/M phases were higher in NP5 than in U87MG (Fig. 4C and Supplementary Fig. 2). To evaluate the NE stress response, NP5 and U87MG were subjected to mechanical stress using the Transwell. Accumulation of γH2A.X at the deformed region of the nucleus was observed in 20% of NP-5 cells, while it was hardly observed in U87MG cells (Fig. 6I,d J). The reduced expression of p21 in NP5 relative to the levels in U87MG was also confirmed by western blotting (Supplementary Fig. 3A). Taken together, these results suggested that sensitivity to NE stress is associated with the stages of cell cycle, and p21 is involved in the regulation of NE repair by modulating cell cycle progression.

### Ineffective NE repair results in apoptotic cell death after mechanical NE stress

**Fig. 7:**
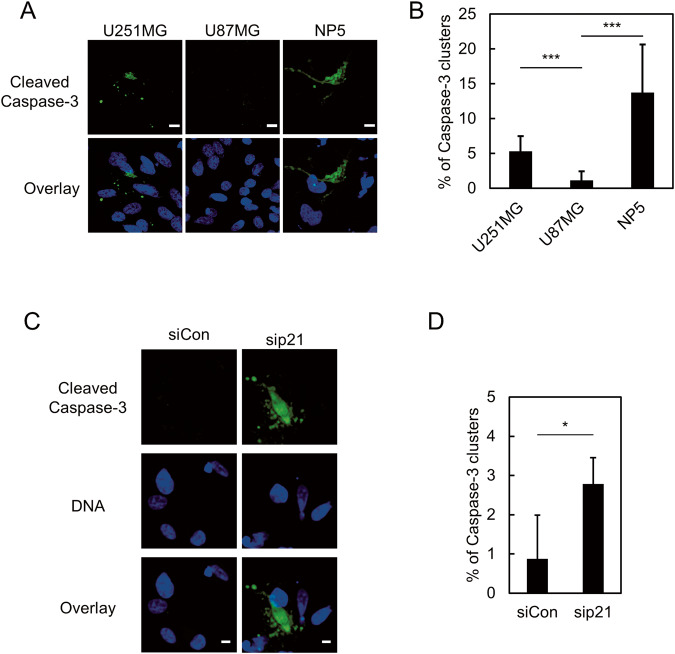
Ineffective NE repair causes apoptotic cell death after constricted migration. **A** Immunofluorescence staining analysis of cleaved caspase-3 (green) of U251MG, U87MG, and NP5 at the bottom side of a Transwell. Overlay indicates merged images with DNA (blue). Scale bars: 10 μm. **B** Quantification of cleaved caspase-3 clusters relative to total cell number in the cells indicated in (**A**). Bars and error bars represent the mean values and standard deviations from three independent experiments. Statistical significance of the differences was determined using Fisher’s exact test. ***p < 0.005. Total cell numbers for each cell line from three independent experiments: *n* = 1045, U251MG; *n* = 701, U87MG; *n* = 357, NP5. **C** Immunofluorescence staining analysis of cleaved caspase-3 (green) of U87MG cells with (sip21) or without (siCon) RNAi of p21 at the bottom side of a Transwell. Overlays represent merged images with DNA (blue). Scale bars: 5 μm. **D** Quantification of cleaved caspase-3 clusters relative to total cell number in the cells indicated in (**C**). Bars and error bars represent the mean values and standard deviations from three independent experiments. Statistical significance of the differences was determined using Fisher’s exact test. *p < 0.05. Total cell numbers for each condition from three independent experiments: *n* = 540, siCon; *n* = 1055, sip21.

Previous studies suggested several pathophysiological consequences of the rupture of the NE, including genome instability, altered gene expression, and senescence [13–15]. Our data revealed that U251MG cells are sensitive to NE stress and prone to a failure to repair ruptured NE. An in vitro assay using microfluidic devices with constrictions suggested that rupture of the NE alone does not cause cell death in cancer cell lines derived from fibrosarcoma or from cervical cancer [3, 4]. However, the deficiency of lamin B1 results in NE rupture concomitant with massive apoptosis of migratory neurons in the developing brain of mice [12]. These findings imply that the consequences of NE stress might be context dependent. Thus, we investigated whether mechanical NE stress causes apoptosis in our model. To achieve this, U251MG, U87MG, and NP5 were plated on a Transwell, and apoptosis was detected by immunostaining using anti-cleaved caspase-3 antibodies. Substantial cleaved caspase-3 signals were observed at the bottom side of the Transwell for U251MG and NP5, whereas for U87MG they were hardly detected (Fig. 7A, B). These findings suggest that mechanical NE stress results in the apoptotic cell death of U251MG and NP5, presumably due to inefficient repair of ruptured NE. To test whether p21 plays pivotal roles in the suppression of NE stress-mediated apoptosis, U87MG cells were subjected to a Transwell assay with and without depletion of p21. Depletion of p21 resulted in a significant increase of the presence of the clusters of cleaved-caspase-3 (Fig. 7C, D). Thus, it is suggested that p21 is indispensable for the suppression of apoptosis induced by NE stress in U87MG cells.

## Discussion

While some of the molecular mechanisms of NE stress response have been clarified, how such processes are affected by the types of cells and/or the cellular environment has not been elucidated. In the present study, we demonstrated that the response to mechanical NE stress varies among cancer cell lines. Furthermore, our data revealed the impact of the cell cycle on sensitivity to mechanical NE stress in GBM. Mechanistically one of the major CDK inhibitors, p21, is indispensable for the resistance to mechanical NE stress and suppresses the consequences of NE stress.

In higher eukaryotes, the NE is completely disassembled at the early onset of mitosis to allow access to the mitotic spindle to the chromosomes [22]. Thus, disassembly and subsequent reassembly of the NE are essential for the progression of the cell cycle. Prior to the disassembly of the NE, many NE components, including BAF and LEM domain proteins, are dissociated from the NE [22]. The change of their localization is mainly regulated by cell-cycle-dependent phosphorylation through several kinases including CDK1 [22, 24, 25, 32]. After the segregation of chromosomes, NE components are recruited to the surface of the chromosomes to establish functional NE. In contrast to the disassembly of the NE, reassembly requires the dephosphorylation of many NE proteins, which is mediated by the inactivation of mitotic kinases [22]. Currently, BAF is known as the earliest accumulating factor to the chromosome among the proteins that are dissociated from the NE prior to NE disassembly [22, 23, 33]. BAF targets LEM domain proteins, leading to further recruitment of ESCRT III complex to coordinate reseal the NE and remove the spindle from chromosomes [22, 34]. Some studies have proposed that the repair of ruptured NE involves a process similar to NE reassembly, featuring sequential targeting of BAF, LEM domain proteins, and ESCRT III to the ruptured site [16, 17, 23]. Thus, it is highly possible that the regulatory mechanisms are also shared between the repair and reassembly of the NE. In this scenario, phosphorylation of the NE repair factors related to the disassembly of the NE will perturb the efficiency of NE repair due to the loss of their capacity to accumulate at the ruptured NE. This can explain why G_2_-phase of cells are more sensitive to NE stress even if the expression of p21 is absent in the entire population of U251MG cells. In this regard, p21 can restore effective NE repair by inhibiting the phosphorylation of NE repair factors. Furthermore, because p21 is depleted in the S phase in U87MG cells, sensitivity to mechanical NE stress is possibly higher at this stage of cell cycle in this cell line as well as in other cancer cells with the same pattern of p21 expression. Dysregulation of the cell cycle is frequently observed in many types of cancer, which potentially impairs the efficient repair of ruptured NE [35]. Our data further support this idea and reveal the link between the cell cycle and NE stress response.

It is possible that cell-cycle-dependent perturbation of NE repair occurs not only in GBM but also in other types of cancers, although we have not observed severe consequences of mechanical NE stress in those cancer cell lines (Fig. 1). Additional investigations will clarify whether the unknown cell-cycle-independent NE repair pathway plays pivotal roles to maintain NE integrity upon mechanical NE stress in those cells. Moreover, further studies are also required to elucidate the significance of apoptosis caused by NE stress in NE stress sensitive cells. Regarding the malignancy of GBM, it is possible that G_1_-phase cells are responsible for the invasion of the GBM in vivo due to their resistance to mechanical NE stress.

In summary, this study revealed that (1) the efficiency of repair of ruptured NE is regulated in a cell-cycle-dependent manner and (2) impaired NE repair caused by dysregulation of cell cycle triggers severe consequences of NE stress in GBM. These novel insights indicate that further detailed investigation of the molecular mechanisms behind the regulation of NE repair should make significant contributions to the development of an innovative therapeutic strategy of GBM.

## Materials and methods

### Cell culture, plasmids, and stable cell lines

U251MG, HT1080, DU145, A549, and U87MG cells were maintained in Eagle’s Minimum Essential Medium (FUJIFILM Wako Pure Chemical Corporation, Osaka, Japan) supplemented with 10% (v/v) heat-inactivated fetal bovine serum (FBS), 1 mM sodium pyruvate, 1X non-essential amino acids, and penicillin/streptomycin at 37 °C in a 5% CO_2_, 95% humidified air atmosphere. MDA-MB468 cells were maintained in Leibovitz’s L-15 Medium (FUJIFILM Wako Pure Chemical Corporation) supplemented with 10% FBS and penicillin/streptomycin at 37 °C in 100% air atmosphere. NP5 cells were maintained in Dulbecco’s Modified Eagle Medium (FUJIFILM Wako Pure Chemical Corporation) supplemented with 10% FBS and penicillin/ streptomycin at 37 °C in 5% CO_2_, 95% humidified air atmosphere. Stable cell lines were established using either transposon or lentivirus. Plasmids for *Sleepingbeauty* based transposon vectors were obtained from addgene. Donor plasmids (pSBbi-Bla: #60526; pSBTet-Pur: # 60507) were transfected together with the expression plasmid for a mutant form of transposase, SB100X (pCMV(CAT)T7-SB100: #34879) using Everyday Transfection (EZ Biosystems, College Park, MD, USA) according to manufacturer’s protocol [36, 37]. A NLS derived from SV40 large T antigen was fused with N-terminus of tdTomato by PCR and resulting fragment was cloned into pSBbi-Bla vector (amplified with PCR) using In-Fusion system (TaKaRa Bio, Kusatshu, Shiga, Japan). cDNA of human p21 was obtained by reverse transcription using ReverTra Ace (TOYOBO, Osaka, Japan) and RNA purified from U87MG. PCR was performed to clone p21 into pmVenus-C1 using *BsrGI*/*EcoRI* sites [9]. Then, resulting mVenus-p21 was amplified with PCR and cloned into pSBTet-Pur (digested with *SfiI*) using In-Fusion system. The stable cell lines expressing mScarlet-BAF were generated by transposon-based vectors indicated above. cDNA of BAF is amplified by PCR using EGFP-BAF plasmid (addgene number: 52962) and cloned into pSBbi-Bla together with mScarlet-I (a gift from Dorus Gadella, addgene number: 98831) [38]. The primers used for construction are listed in Supplementary Table 1. Integrated cells were selected using blasticydin (8 μg/ml) or puromycin (2 μg/ml). The plasmid for FUCCI was obtained from RIKEN BRC (#RDB15454) [30]. To introduce FUCCI reporter set, pCSII lentivirus vector carrying mCherry-hCdt1_(1/100)Cy(-)_ and mTurquoise-hGem_(1/110)_ was transfected into HEK293T cells together with LP1, LP2, and VSV-G plasmids. Resulting lentivirus particles were concentrated using Lenti-X Concentrator (TaKaRa Bio) according to manufacturer’s protocol. Polybrene (8 μg/ml) was supplemented to the medium when cells were infected with lentiviruses.

### Constricted migration assay using Transwell

Constricted migration assay was performed using Transwell with a 3 μm pore size (Corning Incorporated, Corning, NY, USA) as described previously with minor modifications [9]. 1 × 10^5^ cells were plated on a Transwell and cultured for 14–16 h, followed by immunofluorescence staining. For immunofluorescence staining of cleaved-caspase-3, cell were cultured for 40 h.

### EdU labeling and click reaction

To identify S-phase cells, EdU (Jena Bioscience, Jena, Thuringia, Germany) was added into culture medium at 25 μM and incubated for 30 min. Incorporated EdU was visualized by a fluorescent dye through a Cu(I)-catalyzed [3 + 2] cycloaddition (click) reaction, by which terminal alkyne group of EdU is covalently attached with azide group of a fluorescent dye, as described previously with minor modifications [39, 40]. Briefly, cells were fixed with 4% paraformaldehyde (PFA) for 15 min and permeabilized with 0.5% Triton X-100 for 3 min. After three times PBS washes, cells were incubated with the reaction mixture (2 μM picolyl azide Alexa-555 (Jena Bioscience), 20 mM Sodium ascorbate (FUJIFILM Wako Pure Chemical Corporation), 2 mM 2-(4-((bis((1-(tert-butyl)-1H-1,2,3-triazol-4-yl)methyl)amino)methyl)-1H-1,2,3-triazol-1-yl)acetic acid (Jena Bioscience), 1 mM CuSO4 (FUJIFILM Wako Pure Chemical Corporation) diluted in PBS) for 30 min at 37 °C. After three times PBS wash, cells were subjected to the immunostaining if necessary.

### Immunofluorescence staining and nuclear circularity

The cells were grown on coverslips (Matsunami Glass, Osaka, Japan) or Transwell and fixed in 4% PFA for 15 min. After three times wash with PBS, the cells were permeabilized in 0.5% Triton‐X 100 for 3 min followed by blocking with 10% normal goat serum for 60 min. The following antibodies were used as the primary antibodies: anti‐lamin B1 (1:1000; ab16048, Abcam, Cambridge, UK), anti-γH2A.X (1:1000; 05-636, Merk Millipore, Burlington, MA, USA), anti-cyclin B1 (1:200; SC-245, Santa Cruz Biotechnology, Dallas, TX, USA) anti-p21 (1:500; #2947, Cell Signaling Technology, Danvers, MA, USA), anti-cleaved caspase-3 (1:500; #9661, Cell Signaling Technology). Incubation with primary antibodies was performed at 4 °C overnight or 2 h at R.T. For secondary antibodies, goat anti-mouse or anti-rabbit IgG F(ab′)2 fragments conjugated with Alexa 488 or 568 were used (Thermo Fisher Scientific, Waltham, MA, USA). Confocal fluorescent images were acquired using FV1000D (Evident, Tokyo, Japan), FV3000 (Evident, Tokyo, Japan), or Stellaris5 (Leica Microsystems, Wetzlar, Germany). All images were processed through imageJ (National Institutes of Health, Rockville, MD, USA) [41]. The prominent signal of γH2A.X is defined manually with same setting of contrast among a series of the experiments. Obtained images of DNA staining using DAPI were processed to measure nuclear circularity as bellow using imageJ. All the images were subjected to “Gaussian Bluer (sigma 1)” and then Z-stacked with maximum intensity. The binary images were generated using “Threshold (Li, auto)” and subjected to “Fill holes” filter. Then, the nuclear circularity was measured using “Find particles”. Overlapped nuclei were manually excluded.

### Laser irradiation

U251MG and U87MG cells expressing NLS-tdTomato or mScarlet-BAF were plated on the glass bottom dish (Matsunami glass, Osaka, Japan) coated with rat collagen I (Thermo Scientific). Laser irradiation and time lapse imaging was performed using FV1000D. A circular spot with 0.6 μm diameter at the periphery of the nucleus was irradiated with a 405 nm laser at 100% power for 6 s using a SIM light pathway. DNA was stained with SPY650 DNA probe (SPIROCHROME, Thurgau, Switzerland) and visualized with a 635 nm laser. Time lapse imaging was performed with 3 min intervals for 57 min. Obtained images of NLS-tdTomato were processed to measure the ratio of NLS-tdTomato signal in cytoplasm to the nucleus as bellow using imageJ. All the images were subjected to “Subtract Background (Rolling ball radius: 50 pixels, Sliding paraboloid, Disable smoothing), “Gaussian Bluer (sigma 1)”, and then Z-stacked with maximum intensity. Some regions of the nucleus and cytoplasm were manually selected and the mean signal intensity were measured. The signal intensity of cytoplasmic NLS-tdTomato was divided by that of the nucleus, and its highest values during the time course was set as 1.0 for each cell. For the immunostaining of γH2A.X after laser irradiation, the cells were cultured additional 16 h and subjected to immunostaining. All the regions that possibly include irradiated cells were visualized with tile scan using Stellaris5.

### Protein preparation and western blotting

Proteins were extracted from the indicated cells in RIPA buffer (50 mM Tris-HCl (pH 7.5), 150 mM NaCl, 0.1% SDS, 0.5% Sodium deoxycholate, 1% Triton X-100) supplemented with Protease inhibitor cocktail Set V/Phosphatase inhibitor cocktail Set (FUJIFILM Wako Pure Chemical Corporation). The lysates were incubated for 30 min on ice. After centrifugation at 15,000xg for 5 min, the protein concentrations of the supernatants were determined using a bicinchoninic acid assay kit (Thermo Fisher Scientific). Equal amounts of proteins were used for SDS-PAGE. For immunoblotting, the following antibodies were used: anti-cyclin B1 (1:200; SC-245, Santa Cruz Biotechnology, Dallas, TX USA), anti-cyclin E (1:200; SC-247, Santa Cruz), anti-p21 (1:2000; #2947, Cell Signaling Technology), anti‐lamin A/C (1:2000; MABT1341, Merk Millipore), anti-MAN1 (1:10000; TA500794, OriGene, Rockville, MD USA) anti‐β‐actin (1:5000; MAB1501, Merk Millipore).

### Flow cytometry

The cells were trypsinized, washed with PBS once, and fixed with 70% of EtOH for 30 min at 4 °C. After PBS wash, the cells were stained with PI solution (0.15 % Triton X-100, 2μg/ml PI, 50μg/ml RNaseA) at R.T. for 30 min. Cell cycle profile was obtained using FlowJo.

### RNA interference

For knockdown of p21, small interfering (si)RNA targeted to human p21 (AM51331) and negative control (AM4611) were purchased from Thermo Fisher Scientific. siRNA were transfected using Lipofectamine RNAiMax (Thermo Fisher Scientific) as described in the manufacture’s manual. The depletion of p21 was confirmed by western blot 48 h after transfection. Transfection was performed on the top side of the Transwell without the medium at the bottom side. After 12 h Transfection, the medium was added to the bottom side, then the cells were cultured additional 36 h before immunostaining.

### Statistical analysis

Microsoft Excel and R (version 4.0.2) were used via RStudio (version 1.3.1073) for statistical analysis [42, 43].

## Supplementary information

Supplemental Figure 1

Supplemental Figure 2

Supplemental Figure 3

Supplemental Figure Legends

Supplemental Table1

Supplemental Table2

Supplemental Table2 Legend

western-blot-full

## Data Availability

The datasets generated during and/or analysed during the current study are available from the corresponding author on reasonable request.
