# Supplementary material for: Impact of cell cycle on repair of ruptured nuclear envelope and sensitivity to nuclear envelope stress in glioblastoma
Source: Cell Death Discov. 2023 Jul 8;9:233. doi: 10.1038/s41420-023-01534-7 (PMC10329659; doi:10.1038/s41420-023-01534-7)
Supplement: Supplementary file 4 — Supplemental Figure Legends [file 41420_2023_1534_MOESM4_ESM.docx]

**Supplemental Figure 1. Expression level of NLS-tdTomato and accumulation of mScarlet-BAF at the ruptured NE by laser irradiation**

***A***, Visualization of NLS-tdTomato (red) in stable cell lines derived from U251MG and U87MG cells that express NLS-tdTomato. Overlay indicated merged images with DNA visualized with DAPI (blue). ***B***, Quantification of the ratio of signal intensity of NLS-tdTomato to DAPI in the cells shown in (A). Total cell number from two independent experiments: n= 115, U251MG; n= 132, U8MG. Scale bars: 5 μm. ***C***, Accumulation of BAF upon NE rupture induced by laser irradiation of the NE. mScarlet-BAF (magenta) was stably expressed in both U251MG (U251-BAF) and U87MG (U87-BAF) cell lines. Overlay indicated merged images with DNA visualized with SPY650 (green). Arrowheads indicated accumulating mScarlet-BAF.

**Supplemental Figure 2. Cell cycle profile of NP5 cells**

DNA content analysis of NP5 cells using PI staining followed by flow cytometry. A.U.: arbitrary unit.

**Supplemental Figure 3. Western blotting analysis of p21 in GBM cell lines**

***A***, Western blotting analysis of p21 in GBM cell lines, U251MG, U87MG, and NP5 cells. β-Actin was used as a loading control. ***B***, Validation of knockdown of p21 in U87MG. Control (siCon) or p21-targeting (sip21) siRNAs were transfected into U87MG cells. The lysate were prepared 48hrs after transfection. β-Actin was used as a loading control.
