# Supplementary material for: Impact of cell cycle on repair of ruptured nuclear envelope and sensitivity to nuclear envelope stress in glioblastoma
Source: Cell Death Discov. 2023 Jul 8;9:233. doi: 10.1038/s41420-023-01534-7 (PMC10329659; doi:10.1038/s41420-023-01534-7)
Supplement: Supplementary file 7 — Supplemental Table2 Legend [file 41420_2023_1534_MOESM7_ESM.docx]

**Supplemental Table 2. Numbers of the cells used for quantification in this study.**

Numbers for each experiment are represented as “exp1”, “exp2”, and “exp3”. Total number from two (Supplemental Figure 1B) or three independent experiments (except for Supplemental Figure 1B) are represented as “total”.
