# Supplementary material for: Impact of cell cycle on repair of ruptured nuclear envelope and sensitivity to nuclear envelope stress in glioblastoma
Source: Cell Death Discov. 2023 Jul 8;9:233. doi: 10.1038/s41420-023-01534-7 (PMC10329659; doi:10.1038/s41420-023-01534-7)

Figure 4B

Cyclin E/Cyclin B1

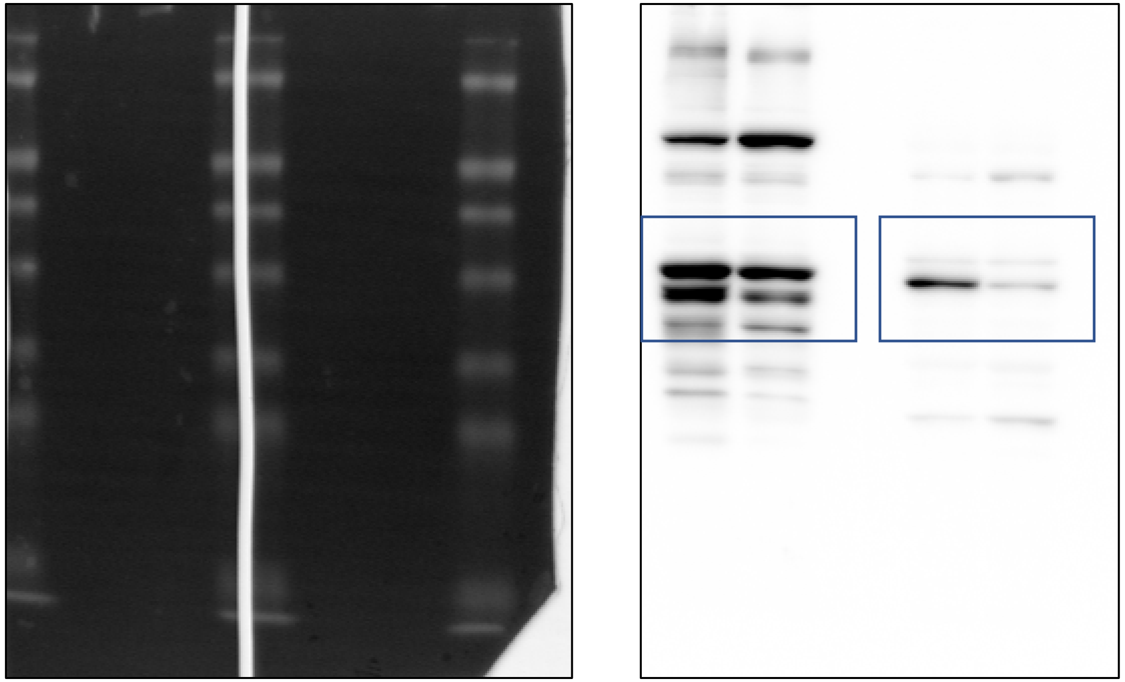

p21

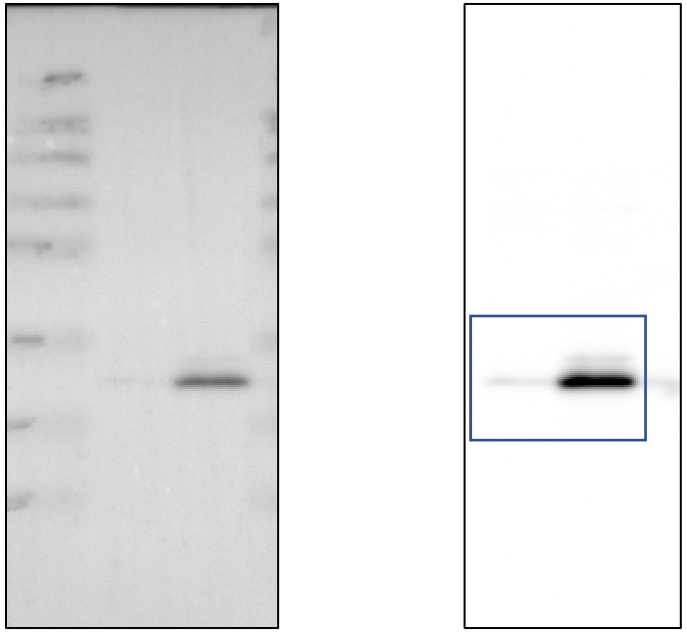

Figure 4B

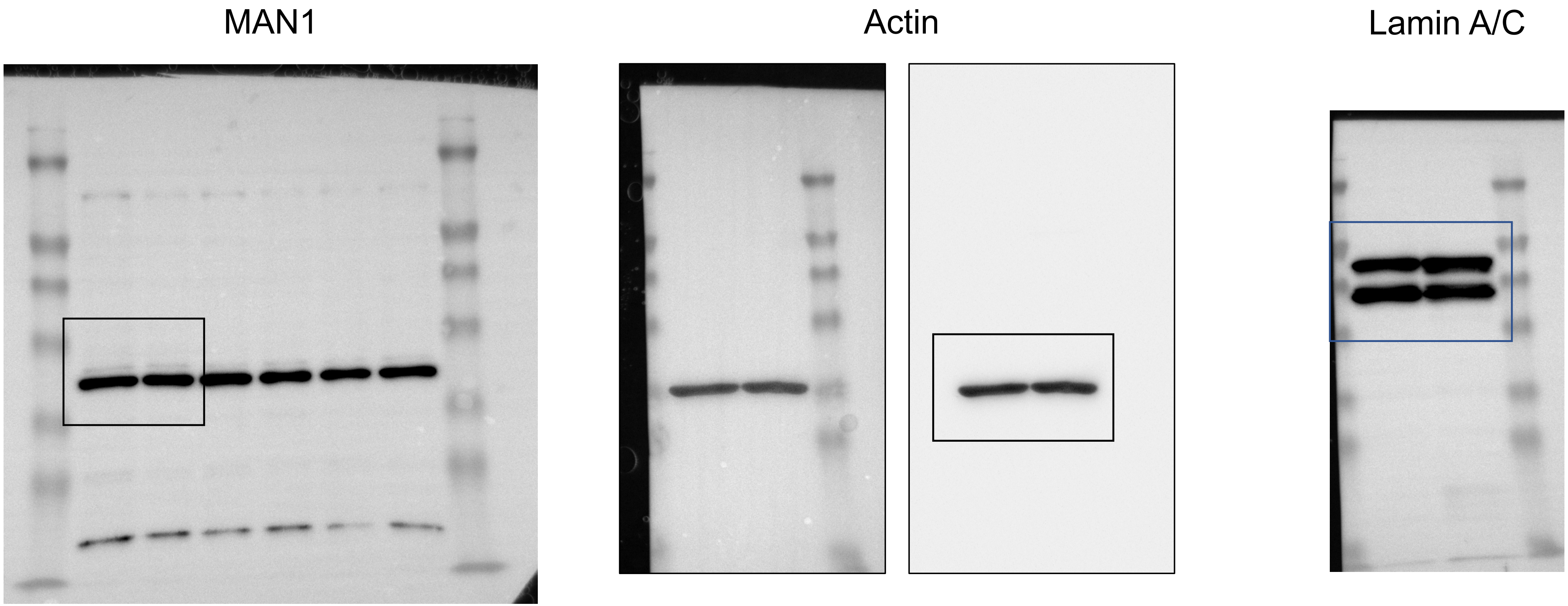

Figure 6A

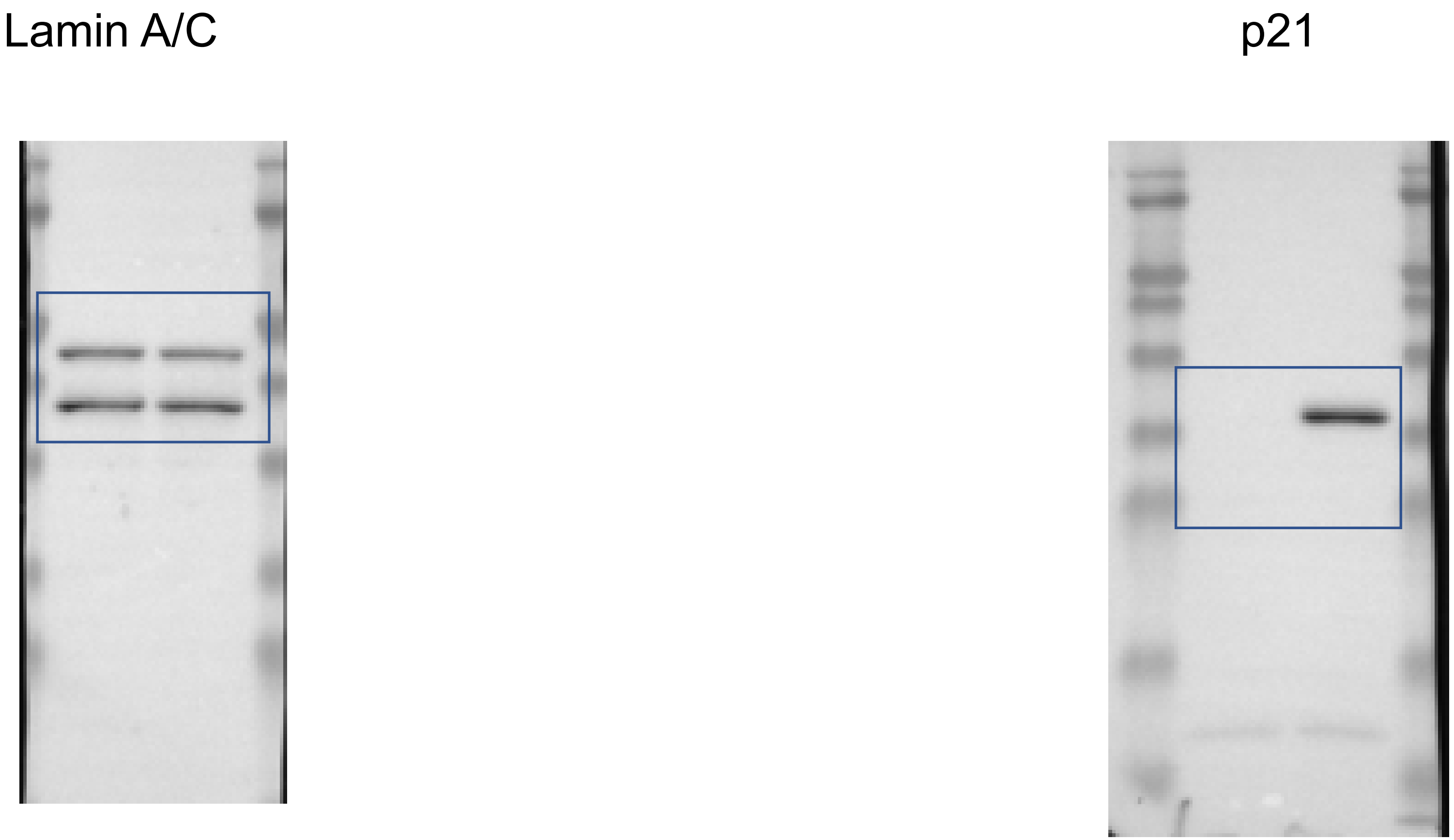

Supplemental Figure 3A

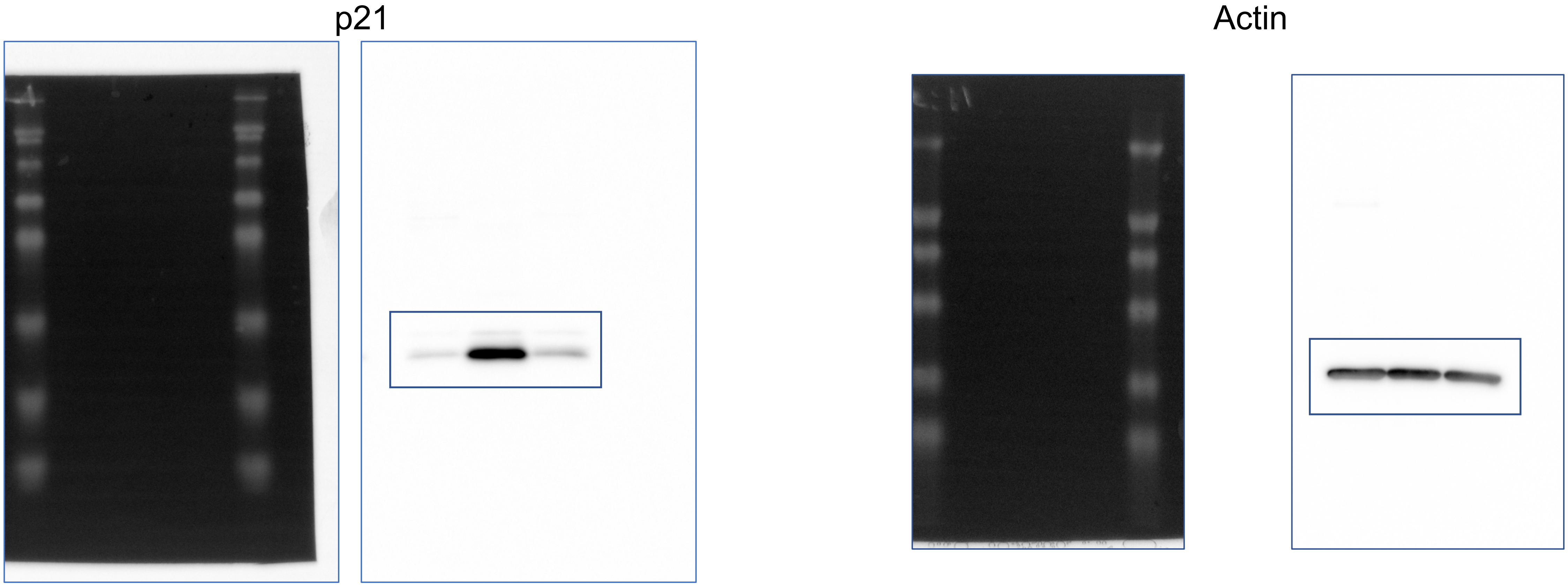

Supplemental Figure 3B

Actin

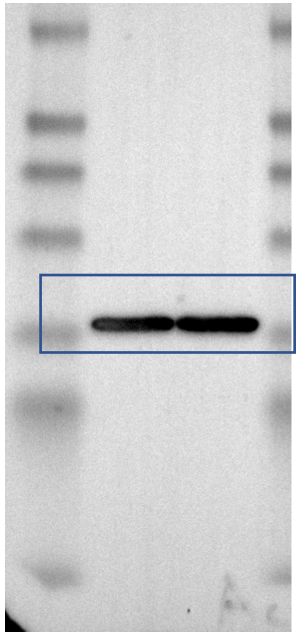

p21

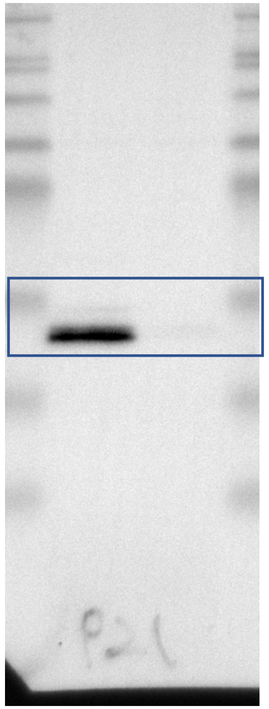

Supplement: Supplementary file 8 — western-blot-full [file 41420_2023_1534_MOESM8_ESM.pdf]
